# Deep mutational scanning and CRISPR-engineered viruses: tools for evolutionary and functional genomics studies

**DOI:** 10.1128/msphere.00508-24

**Published:** 2025-04-24

**Authors:** Mercedes Paz, Gonzalo Moratorio

**Affiliations:** 1 Laboratory of Experimental Virus Evolution, Institut Pasteur de Montevideo 123939, Montevideo, Uruguay; 2 Molecular Virology Laboratory, Faculty of Sciences, University of the Republic 124158 , Montevideo, Uruguay; 3 Center for Innovation in Epidemiological Surveillance, Institut Pasteur de Montevideo 123939, Montevideo, Uruguay; University of Michigan, Ann Arbor, Michigan, USA

**Keywords:** deep mutational scanning, CRISPR-engineered viruses, virus evolution

## Abstract

Recent advancements in synthetic biology and sequencing technologies have revolutionized the ability to manipulate viral genomes with unparalleled precision. This review focuses on two powerful methodologies: deep mutational scanning and CRISPR-based genome editing, that enable comprehensive mutagenesis and detailed functional characterization of viral proteins. These approaches have significantly deepened our understanding of the molecular determinants driving viral evolution and adaptation. Furthermore, we discuss how these advances provide transformative insights for future vaccine development and therapeutic strategies.

## INTRODUCTION

In our 2020 mSphere of Influence article (1), we unfold groundbreaking work in synthetic biology that revolutionized how we study viral genomes, introducing an approach to decode how genetic information drives function, fitness, and evolutionary trajectories. Among these, the seminal study by Coleman et al. (2) demonstrated how engineering codon pair bias in poliovirus genes created a synthetic virus with unchanged protein composition but altered nucleotide sequences. By incorporating host-unfavorable codon pairs, this virus showed reduced replication efficiency and attenuated virulence, while retaining robust immunogenicity, a milestone in designing rationally attenuated viruses.

Building on this foundation, subsequent studies expanded the scope (3–6), applying recoding strategies across diverse viral families. These efforts revealed the intricate links between genome architecture and viral fitness, paving the way for innovative approaches to studying viral evolution and developing vaccine candidates. More recently, researchers have reframed these concepts to engineer RNA viruses using genome-wide recoding strategies aimed at promoting the accumulation of the most detrimental mutations, stop codons, thereby steering viral progenies toward evolutionary “dead ends.” This approach, tested across a spectrum of RNA viruses, including human enteroviruses, influenza, chikungunya, and coronaviruses (7–9), highlights the potential of this strategy to develop live attenuated vaccines and investigate the viral capacity to buffer mutations, known as genetic robustness.

Technological advancements in computational and experimental virology have further catalyzed this progress. Cutting-edge tools now enable precise manipulation of viral genomes, encompassing genome-wide mutagenesis and targeted insertions or deletions to dissect the functional roles of proteins and sequence motifs. This Full Circle review builds upon the existing body of work on viral fitness, focusing specifically on protein-focused methods and their application in deep mutational scanning (DMS) and CRISPR-based engineered viruses. These methodologies are pivotal for dissecting viral mechanisms, developing novel therapeutics, and understanding viral evolution. Here, we highlight recent advances in these two transformative techniques, which represent the next chapter in our understanding of viral fitness. Taken together, these techniques integrate viral genomes with protein function, showcasing the power of these tools in engineering viral genomes. The review will explore the principles behind DMS and CRISPR-based genome editing, discuss their applications in studying viral fitness, and highlight key findings from recent studies. We will also examine the challenges and future directions of these techniques in the context of viral research.

## DMS IN RNA VIRUSES

### Overview and applications

**Fig 1:**
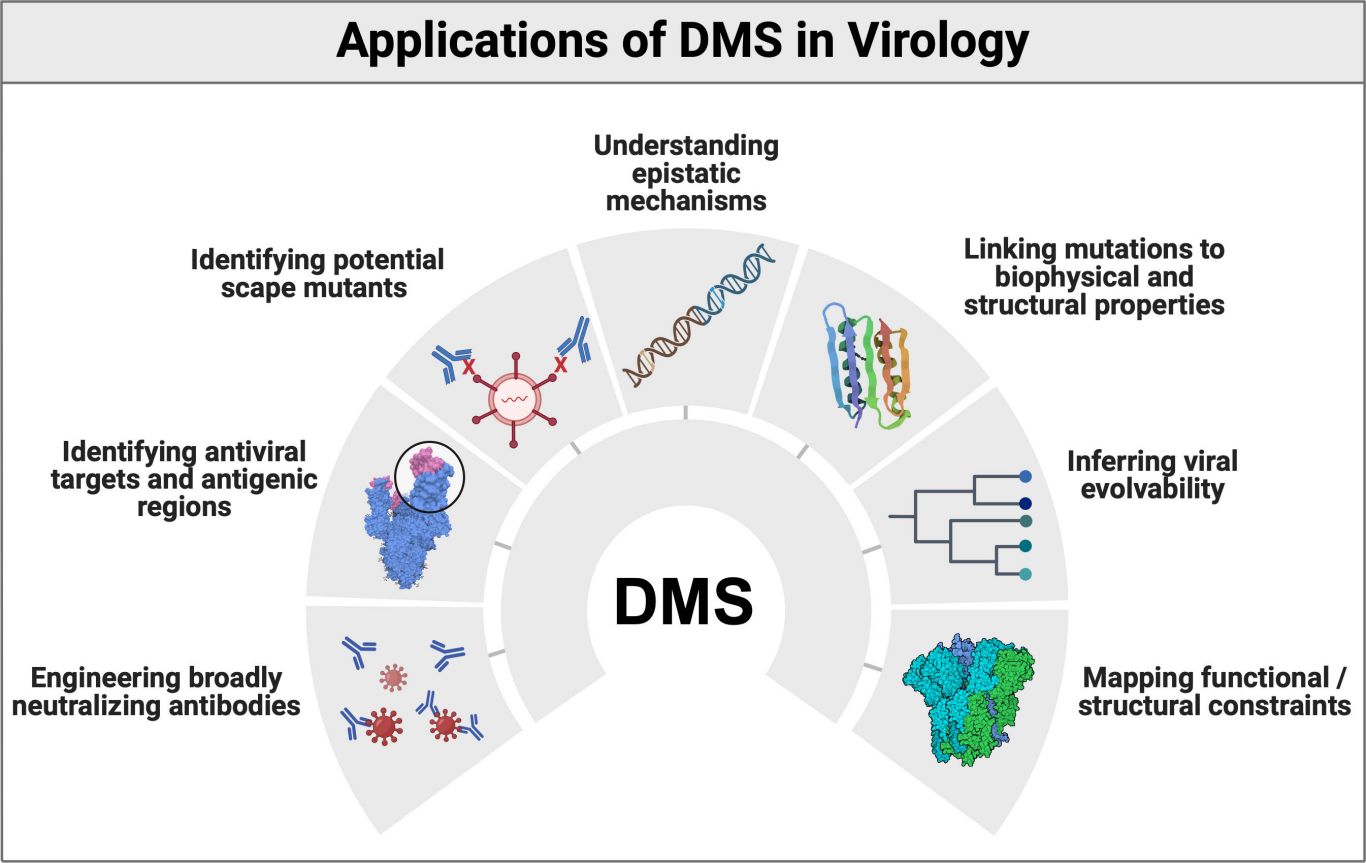
Summary of the main applications of deep mutational scanning in virology. Created in https://BioRender.com.

DMS is a powerful high-throughput technique that systematically investigates how genetic variation translates into phenotypic variation. It enables the creation of fitness maps that detail the effects of nearly all possible nucleotide substitutions within a viral genome or protein sequence. This approach has helped to uncover key molecular mechanisms underlying viral evolution, the roles of non-coding viral sequences, protein function, and resistance to antiviral therapies, among many other applications as summarized in Fig. 1.

Methodologically, a DMS experiment follows at least five key steps. It starts with the design and construction of a large library of sequence variants, which defines the target sequence under study. This sequence can range from an entire viral protein to a particular protein domain, such as active sites or regions involved in important molecular interactions, or even a full viral proteome. The mutational scope is also defined at this stage, including single-nucleotide or amino acid substitutions covering all 19 non-native residues per position, and codon insertions and deletions (known as indels). To generate the mutant libraries, various strategies have been employed. Error-prone PCR has been widely used due to its cost-effectiveness and scalability in high-throughput experiments for introducing random nucleotide substitutions (10–13). However, it is less suitable for experiments in which specific mutations are required and can also introduce biases in the mutation frequency depending on the polymerase used in the PCR (14). Over the past two decades, rapid advances in *de novo* DNA and RNA synthesis have made controlled mutagenesis approaches more accessible, for example, the use of a pool of oligos containing the desired mutations, ensuring precise amino acid or nucleotide substitutions (15, 16) or indels (17).

Following library construction, the mutant proteins must be expressed in a relevant experimental system which is chosen based on the biological question. Infectious virus systems, for example, are used to assess viral fitness by rescuing mutant viral genomes into live viruses and measuring replication efficiency in cells permissive to infections (18–21). Library screening tools used for high-throughput selection of desired traits based on the display of proteins or peptides on the cell surface, like yeast (22–24) or mammalian (16, 25, 26) display systems, and more recently lentiviral or vesicular stomatitis virus (VSV) based pseudoviruses, have been highly beneficial for studying envelope proteins. For example, it was used in studies of the spike (S) protein of SARS-CoV-2 and the hemagglutinin (HA) protein of H5 influenza, to characterize the effects of mutations in phenotypes critical to viral fitness, like viral cell entry, antibody neutralization, and receptor-binding assays (27, 28).

Once expressed, mutants must be filtered to identify beneficial, neutral, and deleterious mutations. For example, in experiments using live viruses, assessing the replicative fitness of mutants after a certain time post-infection or after several rounds of serial passages has been used for evaluating amino acid substitutions in the RNA-dependent RNA polymerase (RdRp) of influenza A virus (IAV) and dengue virus (DENV) (18, 19). Binding affinity assays evaluate how mutations in receptor-binding domains (RBDs) affect interactions with host receptors; for instance, SARS-CoV-2 RBD mutant S proteins are tested for binding strength to human angiotensin-converting enzyme 2 (ACE2) using labeled sorting methods (22, 29). Additionally, neutralization and immune-escape selection experiments can test how mutant envelope proteins influence viral evasion from antibodies, revealing key antigenic sites (23, 29, 30).

Following selection, next-generation sequencing is employed to quantify mutation frequencies before and after functional screening. DNA or RNA from the selected mutant populations is extracted and sequenced using high-throughput platforms. Computational analyses are then used to calculate fitness scores for each variant, categorizing them as deleterious, neutral, or beneficial based on their enrichment or depletion in the selected population (31).

The final step involves interpreting the mutational effects within a structural and evolutionary context. Mapping these changes onto known protein structures using molecular modeling, cryo-electron microscopy, or computational tools like AlphaFold helps predict how mutations alter protein stability and function (15, 16, 32).

Seminal papers published on DMS studies around 2010, originally focused on understanding protein sequence-function relationships. For example, the work by Fowler et al. (33) focused on WW domains, stable, triple-stranded beta-sheet motifs found in signaling and structural proteins that are characterized by their ability to bind proline-rich sequences and evaluated the ligand-binding performance of over 600,000 sequence variants in a single experiment. In another foundational study by Hietpas et al. (34), they developed an approach to systematically generate mutant libraries and measure fitness in a high-throughput manner. Precisely, they mapped the fitness effects of point mutations on a nine amino acid (AA) region of the yeast Hsp90 protein, a chaperone that mediates, directly or indirectly, the folding and activation of 10–15% of all proteins in yeast and humans. Another example of the applicability of this technique is the work of Starita et al. (35), where they used DMS to create a sequence-function map of the U-box domain of the murine E3 ligase Ube4b, characterizing mutations that enhance ubiquitin transfer activity. These works provided the first insights into the distribution and evolutionary weight of different types of mutations, established a tool for identifying mutations that enhance enzymatic activity for biotechnology applications, and demonstrated the potential of DMS in precision medicine, by elucidating the influence of human genome variations on disease susceptibility. As discussed in the following sections, DMS has been used extensively in virology to study viral protein function and immune evasion mechanisms, to contribute to the development of vaccines and antiviral strategies, and to study viral evolution.

### How does DMS shape our understanding of viral proteins?

DMS has greatly been used to map fitness landscapes and constraints on RNA viral proteins. It has helped to identify which AA sites are essential for molecular interactions or enzymatic activity and which ones are more tolerant to mutation, providing a detailed picture of protein function (10, 18, 19, 36–38). Historically, this analysis has been addressed by exploring the grade of sequence conservation of available deposited sequences (39–41). Although sequencing viral genomes from naturally isolated strains has become technically straightforward, only a limited number of viruses have sufficient sequence data available for comprehensive analysis. Furthermore, this approach has been proven to obscure critical residues within highly conserved regions (10, 42). Coupled with corroborative approaches combining experimental validation and computational modeling, DMS has advanced in understanding conserved functional and structural areas, as well as epistatic mechanisms in viral proteins.

Multiple studies have applied DMS to examine viral polymerases and structural proteins. For example, different groups have focused on uncovering functional constraints on the IAV polymerase subunits. Wu et al. (10) combined experimental fitness profiling across 94% of the polymerase A (PA) AA sequence with computational protein structure stability predictions, revealing high inter-type diversity, as numerous functional residues were non-conserved among each influenza virus (A or B) type. Additionally, this integrative methodology successfully distinguished residues critical for viral replication from those essential for protein stability, outperforming conventional approaches like dN/dS analysis (nonsynonymous/synonymous substitution ratio, a method in evolutionary biology to assess selection pressure) and highlighting the limitations of relying solely on sequence conservation or phylogenetic analyses.

Li et al. (19 used DMS to study the mutational landscape of the polymerase basic 1 (PB1) protein. They generated a library covering more than 90% of all possible AA substitutions and quantified their effects on replicative fitness, measured by a viral reconstitution system in mammalian cells. The study revealed that functional and structural constraints primarily mapped to residues involved in molecular interactions with RNA or proteins rather than broader conserved subdomains. In addition to capturing mutational effects on fitness, pairwise competition assays experimentally validated more than 10 mutations, highlighting regions critical for stability and polymerase function. This comprehensive approach demonstrated how DMS can delineate essential regions of RNA virus polymerases while correlating mutational tolerance with evolutionary diversity across natural influenza populations. Similarly, Suphatrakul et al. (18) conducted a DMS study of the nonstructural protein 5 (NS5) protein of DENV, focusing on its roles as an RNA-dependent RNA polymerase and methyltransferase. They performed a saturation mutagenesis experiment targeting nearly all single AA substitutions and assessed their effects on viral replication in mammalian cells. The resulting fitness maps demonstrated high mutational constraint in the conserved RdRp active site but unexpectedly revealed tolerance for select substitutions in universally conserved residues lining the priming loop. These findings suggest potential alternative roles for these residues in regulating polymerase activity. Further integration with structural and biochemical data highlighted the enzymatic versatility of NS5, as certain constrained positions influenced both RNA synthesis and host immune antagonism. This study underscored the importance of combining DMS data with structural modeling to identify residues critical for viral replication and therapeutic targeting. Moreover, recent research on the hepatitis B virus (HBV) polymerase unveiled the mechanism behind cis-preferential reverse transcription (38). They identified conserved prolines near the polymerase’s termination codon that stall ribosomes, effectively tethering the nascent polymerase to its template RNA. Consequently, this interaction ensures cis-preferential RNA packaging and facilitates reverse transcription of the HBV genome.

Structural proteins also play a crucial role in many aspects of viral fitness. They dictate the assembly, infectivity, host-tropism, and survival of viruses. DMS studies on the HA and neuraminidase (NA) proteins of IAV have revealed a clear distinction between antigenic and functional/structural regions (32, 43, 44). Antigenic regions, such as the HA globular head or surface-exposed regions of NA, show high mutational tolerance, enabling immune escape by allowing the virus to evade host immune responses. In contrast, functional domains, including the HA and NA catalytic sites, exhibit low mutational tolerance due to their critical roles in maintaining essential viral functions such as receptor binding and enzymatic activity. These findings highlight the evolutionary constraints imposed by the need to balance immune evasion with functional integrity.

The M1 matrix protein is the most conserved in IAV, leading to the historical assumption that it has a low tolerance for mutations. However, a recent study demonstrated that mutational tolerance varies across the protein, despite the low sequence diversity observed in natural isolates (42). The N-terminal third of M1 exhibited low mutational tolerance, likely due to its critical role in oligomerization and interactions with other viral components. In contrast, the C-terminal two-thirds were more tolerant of AA variations, which structural analysis suggests may result from its disordered configuration, reducing constraints on AA preferences. During the COVID-19 pandemic, multiple studies focused on performing DMS to explore the mutational fitness landscape of the SARS-CoV-2 S protein, shedding light on structural and functional constraints (16, 22, 45, 46). For example, Starr et al. (22) conducted a comprehensive DMS of the RBD of the S protein, measuring the effects of all possible AA substitutions on folding stability and ACE2 binding affinity, which is the main entry point into cells for some coronaviruses including SARS-CoV-2. Their findings showed that whereas most mutations reduced binding or compromised protein folding, some substitutions improved ACE2 binding, most notably, aligning with changes observed in circulating variants. Similarly, Ouyang et al. (16) examined the spike N-terminal domain (NTD) and quantified the impact of thousands of single mutations on protein expression levels, revealing that mutational tolerance was inversely correlated with proximity to interdomain interfaces. Their findings highlighted mutations that enhanced expression without altering antigenicity, offering insights for immunogen design.

### How does DMS advance our understanding of viral evolution?

Over the past decade, DMS has also been instrumental in experimental evolution studies, identifying mutations linked to adaptation and shedding light on evolutionary mechanisms.

Several studies demonstrate the importance of epistasis, which is defined as the interactions between mutations in which one mutation’s effect depends on others' presence. For example, this was done for studying SARS-CoV-2 evolution (29, 47–53), where compensatory mutations (e.g., at the interaction interface of the S protein RBD with the NTD) were demonstrated to reconfigure viral fitness. Taylor and Starr (48) showed that mutations at the RBD of the Omicron SARS-CoV-2 variants BQ.1.1 and XBB.1.5 can induce substantial epistatic effects, where earlier substitutions like N501Y reshaped the mutational fitness landscape, making previously deleterious mutations either neutral or beneficial. Using an infectious system of the whole S protein, Taminishi et al. (51) also revealed epistasis between RBD and NTD mutations, demonstrating compensatory relationships that stabilize overall S protein fitness while enabling immune evasion, such as structural compensation in the NTD for destabilizing RBD mutations observed in Omicron variants BA.1 and BA.2. Recently, epistatic interactions were observed in the Omicron BA.2.86 spike variant, where specific combinations of mutations (e.g., Q493E coupled with L455S and F456L) enhanced ACE2 binding, reversing the deleterious effect of Q493E in previous variants (49). These studies consistently emphasize that epistasis plays an important role in modulating mutational constraints, either relaxing or tightening them based on the genetic and structural context, enabling viruses to navigate evolutionary trade-offs. Moreover, these findings also underscore the importance of investigating epistatic networks across RNA virus families to uncover conserved principles of viral evolution.

DMS has also been applied to investigate how changes in RNA viral proteins influence host tropism, the adaptation of a virus to infect different host species. The work by Thyagarajan and Bloom on the IAV HA protein (32) identified mutations that enable shifts in receptor-binding specificity, highlighting antigenic sites that enhance binding to human-like α2-6-linked sialic acids, a critical determinant for allowing IAV to infect humans. More recently, a comprehensive proteome-wide DMS analysis of coxsackievirus B3 evaluated how over 40,000 nonsynonymous mutations and almost all possible codon deletions in the nonstructural proteins affected its fitness (15). The study revealed considerable variation in mutation tolerance both within and across individual proteins, which is associated with their structural and functional roles. It was found that while many viral proteins face strict functional constraints, nonstructural proteins especially display unique mutational tolerance patterns that may relate to mechanisms of adaptation specific to the host. In another proteome-wide DMS analysis of enterovirus A71, the virus was modified with around 51,000 indels and 41,000 AA substitutions. Studies experimentally examining the effects of indels on viral fitness are scarce. Still, most have indicated that indels are lethal to the virus and can only be tolerated at certain hotspots, which coincide with regions crucial for host recognition and immune interaction. Conversely, the viral proteome shows a higher tolerance for AA substitutions compared to indels, underscoring the evolutionary pressures exerted by this type of mutation and their possible influence on phenotypic diversity (17).

Regarding arboviruses, their dual-host cycle, which involves replication in both invertebrate and vertebrate hosts, imposes a strong purifying selection on the viral genomic determinants required for replication in evolutionarily distant hosts (54). This selective pressure makes it challenging to resolve which determinants are optimal for viral fitness in each host. In a work by Setoh et al. on Zika virus (ZIKV) (55), this was addressed by coupling DMS with fitness measurements across cell types from different organisms (e.g., mosquito cells vs human cells). They characterized conserved residues in the envelope protein’s RBD, two substitutions in the ZIKV E protein, K316Q and S461G, that combined, strongly favored virus replication in mosquito cells. The mutant virus 316Q/461G containing these substitutions was found to be highly attenuated in mammalian cells, organoids, and mice, demonstrating host-specific trade-offs. Similarly, constrained mutational tolerance in critical catalytic sites of nonstructural proteins often links structural stability to host-specific replication efficiency (18). These studies shed light on evolutionary selection, where host-specific evolutionary pressures, whether structural, biochemical, or immune-related, tend to exploit residues or domains that balance adaptability with the preservation of essential functions. They also uncover conserved mutational constraints and hotspots that govern cross-species adaptability in RNA viruses.

### How can DMS identify therapeutic targets?

As a powerful tool for systematically assessing the impact of mutations across viral proteins, several DMS studies have contributed insights that could be relevant to the development of different therapeutic approaches. For instance, mapping viral protein regions related to immune evasion and receptor binding may be possible to enhance the development of neutralizing antibodies (56–58). Moreover, highly conserved regions in viral proteins could be ideal targets for therapeutic and preventive strategies against RNA viruses. For example, these regions have the potential to be used for the development of broadly neutralizing antibodies that focus on conserved epitopes in envelope glycoproteins, to ensure cross-strain efficacy, such as those reported to target a discrete membrane-proximal anchor epitope of the HA stalk domain in IAV (59). In addition, antivirals targeting critical enzymatic or functional sites in these conserved regions found by DMS experiments could have the potential to effectively inhibit viral replication, offering a promising alternative to the limited number of effective vaccines available to combat diverse RNA viruses (44).

A promising alternative approach to monoclonal antibodies and antivirals, which has capitalized on DMS studies identifying mutational rigid and conserved receptor-binding regions, involves the designing of soluble decoy receptors that mimic the virus-binding regions of host receptors. In a series of studies, a research group used deep mutagenesis of the ACE2 receptor to first engineer a decoy receptor sACE22.v2.4, with enhanced affinity for the SARS-CoV-2 S protein (60). The decoy receptor, containing three mutations (T27Y, L79T, and N330Y), showed a 35-fold increase in binding affinity and neutralized SARS-CoV-2 at levels comparable to high-affinity monoclonal antibodies. In the first study, the engineered receptor was demonstrated to be highly soluble, stable, dimeric, and catalytically active, with broad neutralization capabilities against both SARS-CoV-1 and SARS-CoV-2. In a second study (61), the researchers explored the potential for viral resistance to sACE22.v2.4 and found that no mutations within the RBD of SARS-CoV-2 redirected specificity toward the wild-type ACE2 receptor, suggesting that resistance would be rare. In another collaborative work, the *in vivo* efficacy of a derivative of the sACE22.v2.4 decoy fused with an IgG1 constant region (sACE22.v2.4-IgG1) was evaluated in K18-hACE2 mice (62). This work proved that sACE22.v2.4-IgG1 exhibited tight and persistent binding to SARS-CoV-2 and several variants of concern, and notably, that the decoy significantly reduced viral entry, lung vascular hyperpermeability, acute respiratory distress syndrome, and mortality in mice infected with both the WA-1/2020 and P.1 VOCs. These works highlight the promising therapeutic potential of an engineered decoy receptor *in vivo*. However, its clinical application is still being studied due to the possible short serum half-life, which may compromise its practical use. A recent study on the engineered decoy receptor sACE22.v2.4-IgG1 points out that sialylation is a vital factor in enhancing the *in vivo* efficacy of sACE2 decoy receptors and stresses the importance of glycosylation modifications to improve their pharmacokinetics (63).

### Virus-driven crispr screens to identify host factors modulating viral replication

#### Overview

The pioneering work of Mojica et al. (64) and Barrangou et al. (65) demonstrated CRISPR-Cas9 as the adaptive immune mechanism of bacteria and archaea to fight foreign genetic material such as plasmids and phages. In prokaryotes, this system specifically recognizes and binds DNA through CRISPR RNA (crRNA), guiding Cas proteins to identify and cleave exogenous DNA with the assistance of trans-activating CRISPR RNA (tracrRNA). Subsequently, the groups of Charpentier and Doudna (66) combined crRNA and tracrRNA into a single-guide RNA (sgRNA), which more efficiently facilitated Cas9’s genome-editing role *in vitro*. This groundbreaking discovery, which earned them the Nobel Prize in Chemistry in 2020, transformed our ability to investigate gene functions. In virology, the CRISPR-Cas9 system has widely been used to study virus-host interactions through whole-genome screening, using vector-based sgRNA libraries targeting the whole coding host genome, identifying host factors essential for virus replication (67–72).

**Fig 2:**
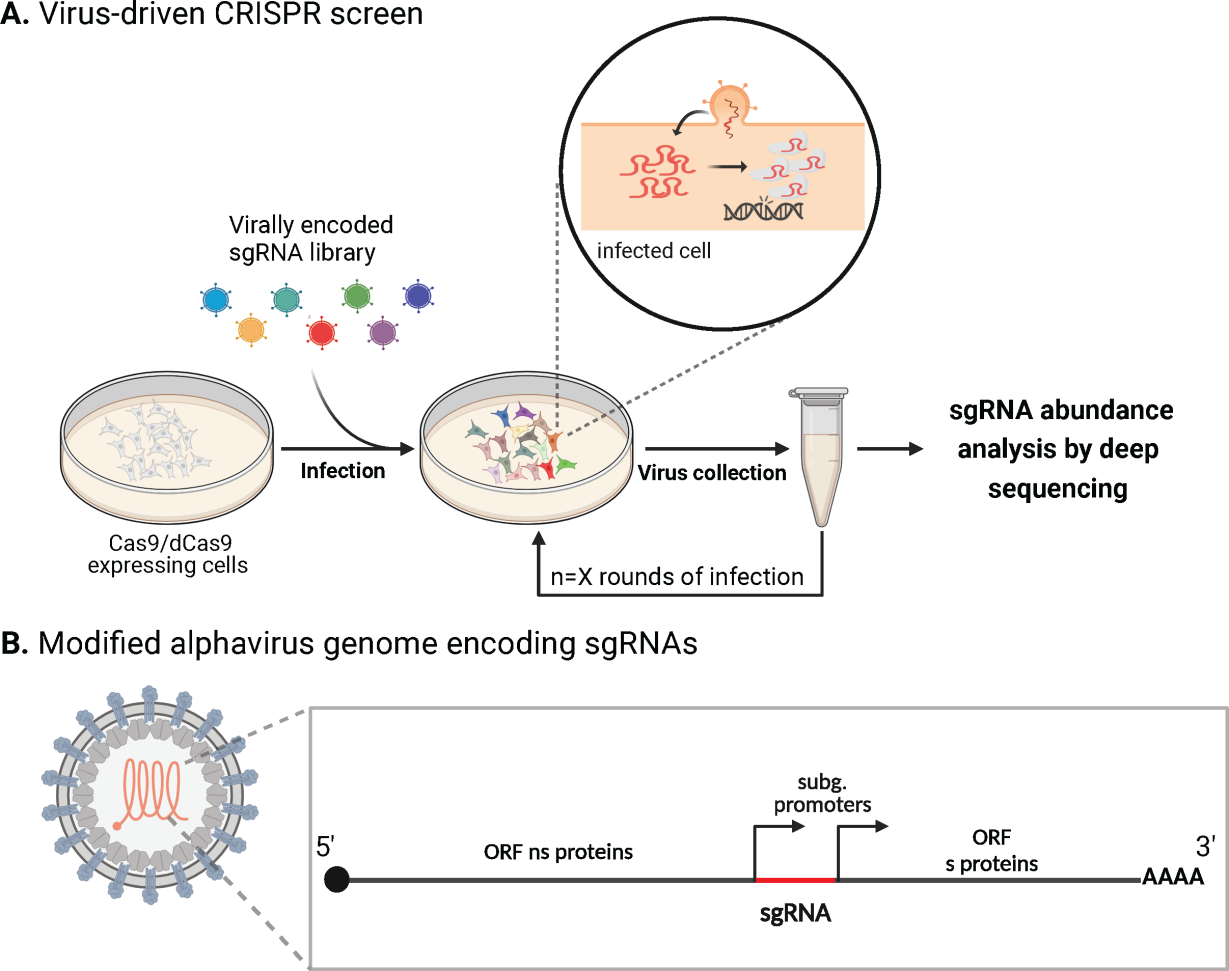
Virus-driven CRISPR screens. (**A**) Overview of a virus-driven CRISPR screen. (**B**) Potential strategy for the development of alphaviruses encoding sgRNAs. Open reading frame (ORF), nonstructural (ns) proteins, subgenomic promoters (subg. promoters), structural (**S**) proteins. Created in https://BioRender.com.

Until just a couple of years ago, most multiplexed pooled CRISPR screens in the context of infection were based on cell viability as their readout, leading to the discovery of cellular genes that allow cells to resist infection. Although extremely powerful, cell viability approaches predominantly reveal hits that block the early steps of viral replication, specifically entry, limiting the breadth of captured host factors. As reviewed in the following section, various groups have developed a novel screening system based on CRISPR-engineered replicating viral systems to expand the breadth of discoverable functional factors and increase sensitivity. In this platform, sgRNA libraries are expressed directly from the viral genome, enabling the direct follow-up of virus propagation throughout the infection cycle. Upon infection of a cell expressing the Cas9 protein, the sgRNA encoded by the virus is released and alters the expression of cellular genes. This gene alteration affects the replication kinetics of the virus, where the sgRNA levels serve as a direct readout for viral propagation (Fig. 2A). In this way, sgRNAs that target genes that are essential or restrict virus propagation are expected to be underrepresented or enriched, respectively, in viruses budding from Cas9-expressing cells. Additionally, since thousands of viral genome copies are synthesized during each replication cycle, the signal is naturally amplified, making this screening approach highly sensitive and, potentially enabling the detection of even small positive and negative effects on viral propagation. This methodology surpasses conventional CRISPR screens by offering greater specificity, resolution, and mostly, the ability to profile later stages of the infection cycle, paving the way for novel antiviral strategies.

### How can viruses be engineered to conduct CRISPR screens?

This novel approach was first applied in a study to identify interferon-stimulated genes against HIV (73). In this study, the researchers used lentiviral genomes encoding sgRNAs packaged into HIV virions, enabling the delivery and expression of Cas9 and sgRNA in target cells. Knockout cells were pooled, and deep sequencing of CRISPR RNAs in newly budded virions was used to assess HIV replication efficiency across thousands of genes in a heterogeneous population. The study identified antiviral factors like MxB, TRIM5α, IFITM1, and Tetherin, with sensitivity varying by viral strain. Additionally, host dependency factors such as CD169, SEC62, and TLR2 were shown to support HIV replication in THP-1 cells. In another study on HIV (74), the researchers employed a library of over 1,500 replication-competent HIV-1 constructs, each expressing a single sgRNA targeting more than 500 cellular genes, to identify antiviral restriction factors across the entire HIV-1 replication cycle. Passaging in CD4^+^T cells enriched sgRNAs targeting several key genes, including GRN, CIITA, EHMT2, CEACAM3, CC2D1B, and RHOA, by over 50-fold. Additionally, the technique identified IFI16 as a restriction factor specifically targeted by the HIV-1 accessory gene *nef*. Functional validation in cell lines and primary CD4^+^T cells confirmed that these factors impact various stages of the HIV life cycle, including entry, transcription, release, and infectivity.

Recently, Finkel et al. (75) introduced virus-encoded CRISPR-based direct readout screening (VECOS), a system leveraging human cytomegalovirus (HCMV) engineered to express sgRNAs targeting the human coding genome. This was achieved by cloning the HCMV genome into a bacterial artificial chromosome system coupled with a Gateway cloning system to streamline the process of modifying the viral genome to encode a robust sgRNA library. Primary human foreskin fibroblasts (HFFs) were employed as the experimental model due to their permissiveness to viral replication. Sequential infections were performed by harvesting viral supernatants from infected HFFs and transferring them to fresh cultures to propagate the virus over multiple passages. To quantify changes in sgRNA abundance across passages and identify host factors critical for distinct stages of the HCMV infection cycle, the researchers extracted viral DNA after each sequential passage from cell lysates and culture supernatants. The abundance of sgRNAs encoded within the viral genome was then analyzed by PCR and high-throughput sequencing. This experimental approach demonstrated that RRM2, a key enzyme in dNTP production, is a critical host factor for HCMV replication, as its depletion severely impaired viral propagation and DNA synthesis. This finding opens up a new therapeutic potential for combining RRM2 inhibitors with existing anti-HCMV treatments, such as ganciclovir, to enhance antiviral efficacy. Importantly, the study demonstrated the unique strengths of VECOS in identifying a broader range of host factors, including those critical for non-lethal stages of infection, which are often missed by traditional survival-based CRISPR screens. They also provide evidence of the poorly understood functions of ARL6IP6 and its importance in viral propagation, demonstrating that ARL6IP6 plays a critical role in the late stages of the HCMV infection cycle, likely contributing to virus assembly through its association with ER-derived structures.

Similarly to the group above, King et al. (76) developed a novel fitness-based screen named transcriptional regulation by pathogen-programmed Cas9 (TRPPC), which uses IAV to deliver single guide RNAs (sgRNAs) that program CRISPR activation or CRISPR inhibition of cellular genes exploiting the virus’s ability to express non-coding RNAs. They modified the influenza virus nonstructural (NS) gene, which normally encodes two proteins, nonstructural protein 1 (NS1) and the nuclear export protein, by altering its overlapping reading frames into non-overlapping ones, separated by an artificial intron. This created a “split NS” system that allowed the expression of non-coding RNAs without disrupting viral protein production or replication. They incorporated a microRNA (miRNA) encoded within this artificial intron, which would be processed by the host’s Drosha nuclease in the nucleus, along with a sgRNA downstream of the miRNA-124, releasing the sgRNA from the viral genome. After several rounds of selection of the virus-encoded sgRNA library in A549 cells, the screen identified the protein TREX1, a cytoplasmic DNA exonuclease, as a key pro-viral factor, as it degrades cytoplasmic DNA to prevent immune activation, thus aiding influenza replication. This methodology also allowed the creation of a ranking of host factors that affect IAV viral replication, providing valuable insights into host-pathogen interactions.

### Challenges and future directions

Despite their potential, the mentioned methodologies face challenges that create opportunities for refinement to address research gaps and limitations. A technique like DMS produces a big volume of data, and the main challenge remains in analyzing and making sense of it to predict the effects of mutations. Machine learning offers a powerful approach for these methods that require the analysis of vast amounts of data and exhaustive experimental testing. For example, automated viral infectivity assays utilizing convolutional neural networks have been used to predict infection phenotypes from microscopy data, improving speed and precision in virus characterization, which could be applied for a more efficient analysis of viral variants (77–79). Moreover, machine learning has also been used to predict the functional outcomes of millions of untested mutations from DMS data sets, helping researchers to identify and filter promising variants for further study and reduce the need for exhaustive experimental testing (80–82). This approach could fill gaps in DMS by enabling real-time predictions of mutation effects in complex protein landscapes and expand the ability to explore larger mutational spaces with high accuracy and fewer laboratory resources. Another major challenge, shared by both CRISPR screening and DMS, is that they rely on cell culture as their experimental system, which can influence the interpretation of mutational effects. While these systems are used primarily for their ability to facilitate high-volume infections and vigorous viral replication, they may not fully capture the complexities of *in vivo* scenarios, where factors such as tissue-specific interactions and immune responses may play a significant role. For instance, research teams employing DMS on the same viral protein have reported inconsistent results across diverse cellular environments (42, 83). The authors discuss that discrepancies can largely be attributed to differences in viral strains, cell types, and experimental methodologies, but cannot rule out factors like population bottlenecks and epistatic interactions (42). Therefore, any conclusions drawn should be contextualized within the specifics of the viral strain and experimental setting.

Regarding DMS, although several studies have identified conserved functional regions in RNA virus proteins, such as the flavivirus NS5 or influenza PB1 polymerases (18, 19), systematic cross-family comparisons of mutational constraints are scarce. Current research mostly focuses on specific viral targets or homology-based analyses, lacking comprehensive data sets that consolidate DMS mappings across diverse RNA virus families to identify conserved functional or structural “hotspots.” This gap may largely arise from inconsistencies in experimental models and the lack of standardized fitness metrics across studies. Future research could expand DMS efforts to homologous proteins across major RNA viral families, targeting universal antiviral sites such as RdRp enzymatic cores and conserved glycoprotein domains. Such integration could inform the design of broad-spectrum inhibitors targeting residues less prone to mutational escape, paving the way for cross-family antiviral strategies, like the development of pan-viral vaccines to combat diverse RNA viruses. To support this idea, initiatives such as MaveDB (84), an open-source repository for sharing and analyzing DMS data sets, and the Atlas of Variant Effects Alliance (https://www.varianteffect.org/) have been established to foster collaboration and enhance the utility of mutational scanning research (85).

As part of this section discussing future directions, we hypothesize that alphaviruses, with their positive-sense, single-stranded RNA genomes that replicate in the cytoplasm, offer a distinct opportunity for exploration as a possible advancement in CRISPR/Cas9 technology. These viruses exhibit unique characteristics compared to previously employed viral systems. Specifically, the potential development of replication-competent Mayaro virus constructs encoding sgRNAs represents an innovative approach worth investigating. This idea is grounded in the capacity that alphaviruses have to tolerate the insertion of exogenous coding and non-coding sequences downstream of their nonstructural genes, expressed via a subgenomic promoter (Fig. 2B). To facilitate the release of non-coding RNAs like sgRNAs during replication, these sequences can be positioned downstream of a duplicated subgenomic promoter, followed by a self-excising ribozyme (e.g., HDV) or a miRNA sequence, as demonstrated in prior studies (76, 86). Nevertheless, RNA viruses face additional complexities, including high mutation rates and genomic instability, which increase the risk of evolutionary escape and may compromise embedded sgRNA functionality. Optimizing experimental designs and methodologies will be essential to make CRISPR-based tools broadly applicable across multiple RNA viral families. In addition, given the recent development of this technology, establishing robust reproducibility standards is critical to ensure consistent and reliable outcomes across viral strains. Variability in experimental protocols, such as delivery methods, cell lines, and infection conditions, affects sgRNA targeting efficiency and the functional relevance of identified host factors. Addressing these issues will require standardized sgRNA libraries and multi-laboratory validation studies to benchmark findings and enhance reproducibility.

## CONCLUDING REMARKS

In this Full Circle review, we highlight how DMS and CRISPR-engineered viral systems serve as essential tools in virology, enabling researchers to map viral fitness landscapes, identify drivers of evolution, and explore host-virus dynamics. DMS has revolutionized the study of viral proteins, allowing us to create fitness maps that detail the effects of nearly all possible nucleotide substitutions within a viral genome. Combining experimental techniques with computational tools such as modeling and machine learning has provided deeper insights into mutations, protein functions, and epistatic interactions to unveil viral evolution. Global collaboration is crucial, with open-access repositories like MaveDB enabling data sharing to address broader challenges like assessing mutational landscapes across viral families or identifying pan-antiviral targets. The newly developed strategy, virus-driven CRISPR screenings, focuses on the role of host factors in viral replication, uncovering novel host-viral mechanisms of interaction and revealing new therapeutic targets. It represents a significant advancement over earlier conventional CRISPR screening, addressing limitations such as profiling later stages of the infection cycle. As this technology develops, new experimental designs and methods will broaden the application across multiple viruses, revealing new insights into viral-host dynamics and offering promising leads for antiviral development.

Looking ahead, the convergence of high-throughput technologies, artificial intelligence, and viral genome engineering will unlock an era of unprecedented precision in virology. This multidisciplinary approach will not only accelerate our understanding of viral biology but will also lead to personalized, adaptive treatment regimens, representing a new frontier in the fight against viral infections.
